# Giant Thrombosis at Left Anterior Descending Artery Aneurysm in a 10-Year Old Boy with Granulomatosis with Polyangiitis

**DOI:** 10.1155/2020/3417910

**Published:** 2020-04-19

**Authors:** Ehsan Aghaei Moghadam, Nahid Aslani, Helia Mojtabavi, Farnoosh Larti, Azin Ghamari, Vahid Ziaee

**Affiliations:** ^1^Department of Pediatrics, Tehran University of Medical Science, Tehran, Iran; ^2^Children's Medical Center, Pediatrics Center of Excellence, Tehran, Iran; ^3^Department of Pediatrics, Isfahan University of Medical Science, Isfahan, Iran; ^4^Growth and Development Research Center, Tehran University of Medical Science, Tehran, Iran; ^5^Pediatric Rheumatology Research Group, Rheumatology Research Center, Tehran University of Medical Sciences, Tehran, Iran

## Abstract

Granulomatosis with polyangiitis (GPA), necrotizing vasculitis of small and medium-sized vessels, is traditionally believed to mainly affect respiratory tract with additional focal kidney involvements as its primary manifestations with a relatively rare annual incidence rate of 20-50 cases per million. Six percent of the affected cases have cardiac involvements; among which, aneurysms comprise the lowest penetrance. By this paper, we aim to cast light on clinical diagnostic and treatment methods of a rare case presentation, a 10-year-old male GPA patient, diagnosed with massive thrombosis at his coronary artery aneurysm. GPA should be considered as differential diagnosis of prolong fever and coronary aneurysms in adolescents.

## 1. Introduction

Granulomatosis with polyangiitis (GPA) or Wegener's granulomatosis as a necrotizing vasculitis of small and medium-sized vessels is traditionally believed to mainly affect respiratory tract with additional focal kidney involvements as its primary manifestations with a relatively rare annual incidence rate of 20-50 cases per million. Multiorgan involvements including renal 18%, gastrointestinal tract 10-12%, cardiac 6%, and neurologic 5% have been described recently by the literature. Despite the low incidence, uncommon organ involvements are severe and could be fatal in case remain untreated [1–3].

Definitive granulomatosis with polyangiitis (GPA) diagnosis lies on fulfilling three out of six following criteria: compatible histopathology with granulomatosis inflammation within the wall of an artery or in the perivascular region, oral or nasal chronic involvement, laryngo-tracheobronchial stenosis, radiographic findings suggestive for pulmonary lesions, positive enzyme-linked immunosorbent assay (ELISA) test for antineutrophil cytoplasmic antibodies (ANCAs), and renal involvement [4]. The most common clinical and laboratory features present at the time of diagnosis were constitutional symptoms, upper and lower respiratory tract, mucosa, and skin and musculoskeletal involvement. In the pediatric population, the disease occurs in the second decade of life, with a female preponderance [5].

Cardiac complications of GPA include pericarditis, coronary arteritis, aortic regurgitation, aortic valvular lesions simulating endocarditis, heart block, and aneurysms [6].

By this paper, we aim to cast light on clinical diagnostic and treatment methods of a rare case presentation, a 10-year-old patient, diagnosed with massive thrombosis at his coronary artery aneurysm.

## 2. Case Presentation

The patient was a ten-year-old boy who had been referred to children's medical center as a pediatric center of excellence for diagnostic and therapeutic evaluation due to persistent fever associated with upper respiratory infection symptoms and lower limb pain. In the previous medical center, he was treated with antibiotic as bacterial pneumonia that resulted in poor clinical response. After which, antituberculosis regimen was initiated mainly based on endemic region ethnicity for the mycobacterium and cavitary pulmonary lesion on radiographic studies. He also had an episode of perforated otitis media with effusion (OME) with additional mastoiditis receiving ventilation tube as the treatment. Alongside the disease course, he experienced antibiotic-resistant sinusitis for 2 months requiring surgical treatment. By this point, the diagnosing team focused on immunologic work up; hence, his recurrent infections which revealed our child had a competent immune system. It is noteworthy that the patient and his family reside in a different city than our clinic so not all the therapy happened under our supervision.

**Figure 1:**
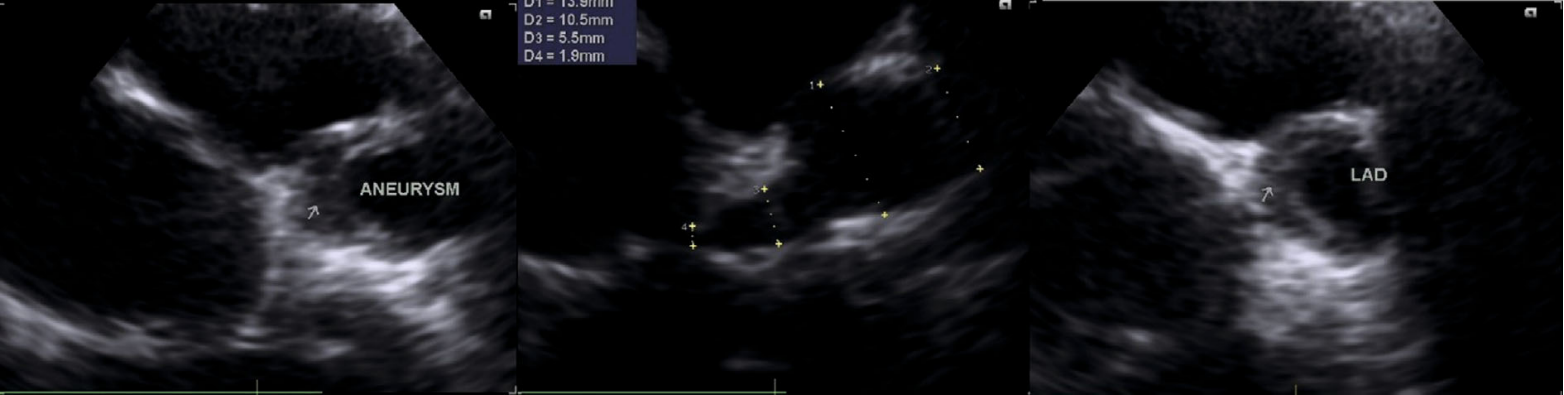
Short axis view (SAX) of transthoracic echocardiography (TTE) in our patient shows ectasia at left anterior descending (LAD) artery and huge aneurysm with large circular nonorganized thrombosis occupying the majority of the aneurysm, narrowing the internal lumen of the artery.

After 6 months, he was referred to Children's Medical Center, Pediatric Center of Excellence in Tehran as a tertiary referral center for more diagnostic evaluation and treatment. There was no arthritis in the lower extremity examination, but due to the difference in lower limb size, an evaluation for thrombosis was performed that was suggestive of deep vein thrombosis (DVT). Due to prolonged fever and increased inflammatory markers such as elevated erythrocyte sedimentation rate (ESR = 105), C-reactive protein (CRP = 95), leukocytosis (WBC = 17,600), and thrombocytosis (Plt = 635,000), pediatrics rheumatologists were consulted for the possibility of rheumatologic diseases and collagen vascular disorder. Transthoracic echocardiography was performed showing diffuse ectasia and dilation with huge aneurysm in the left anterior descending artery (LAD) with the size of 14 mm (Figure 1). Although, there was no other clinical signs in favor of Kawasaki disease other than prolonged fever, due to the high inflammatory markers and age of patient and the possibility of atypical neglected atypical Kawasaki disease or other rare vasculitis.

Other positive findings include antinuclear antibody (ANA) with titer of 1/640 and coarse speckled pattern in indirect immune fluorescent assay (IIF) and antineutrophil cytoplasmic antibodies (ANCAs) with perinuclear pattern. Regarding thrombosis, an assessment for antiphospholipid antibodies and antiphospholipid syndrome was performed, which was negative. Serum immunoglobulins, complements, and nitroblue tetrazolium were normal.

**Figure 2:**
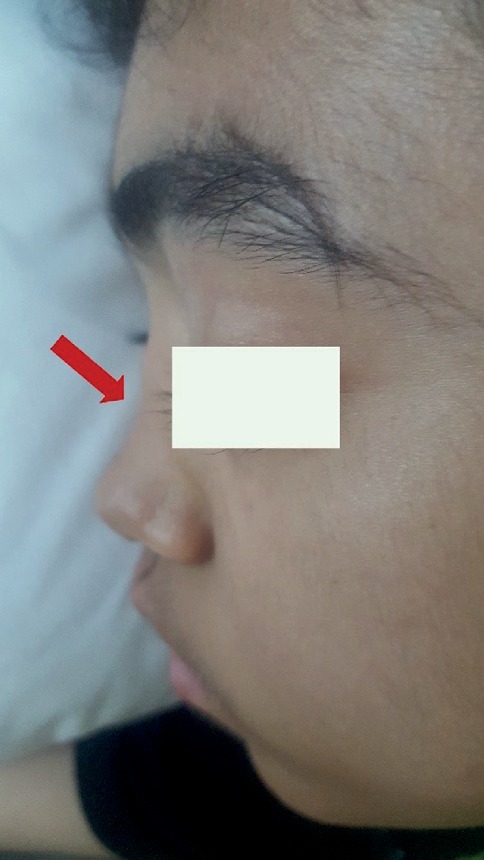
Saddle nose in our patient with granulomatosis with polyangiitis.

Finally, based on the history of severe upper airway infection, pulmonary cavitary lesion, saddle nose (Figure 2), elevated ESR and CRP, positive ANA and ANCA with perinuclear pattern (P-ANCA), granulomatosis with polyangiitis (Wegener granulomatosis) was diagnosed for this patient. Initial treatment as induction therapy was methylprednisolone pulse (30 mg/kg/day for 3 days/monthly) and cyclophosphamide pulse 750 mg/m2/monthly for 6-month duration. Oral medication was administered with prednisolone (1/mg/kg/day) and mycophenolate mofetil (1,200 mg/m2/day). An additional aspirin (5 mg/kg) plus warfarin (0.1 mg/kg) was administered to prevent further thrombotic events by maintaining the INR level at the range of 2-3.

**Figure 3:**
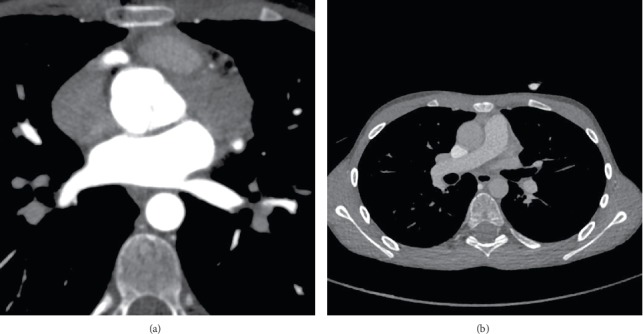
(a) High-resolution, ECG-synchronized computed tomography of the heart with attention to the coronary arteries showed left main coronary ectasia (4.2 mm), fusiform aneurysmal dilation measured 19 mm length, and14 mm transverse diameter with partial thrombosis in the lumen measured 17 × 10 mm in proximal segment of left anterior descending artery (LAD) and aneurysmal dilatation in proximalpart of left circumflex (LCX) in 5 mm diameter. (b) A cavity formation in the left lung apex measure 36 × 27 × 21 mm and some fibrotic and atelectatic bands in bases of both lung.

On a follow-up, transthoracic and transesophageal echocardiographic evaluation, large circular nonorganized thrombosis occupying the majority of the aneurysm was noted that was confirmed by CT angiography despite the anticoagulation prophylaxis (Figure 3). To target the thrombosis, high dose of methylprednisolone and 10 mg/kg of heparin were applied initially, then warfarin was added subsequently to maintain INR level at 3-4. In interventional cardiology consult suggested medical therapy without any intervention due to high risk thrombotic complications.

In a longtime follow-up, he is under maintenance therapy for GPA (with low-dose prednisolone, mycophenolate mofetil, and antithrombotic therapy for 1.5 years without any flare up of underlying disease, but serial transthoracic echocardiography showed minimal improvement in the size of aneurism and thrombosis).

## 3. Discussion

GPA is one of the most common necrotizing vasculitides. The prevalence of the disease is 3 in 100,000 populations with male to female ratio of 3 : 2, and peak incidence is at the age of 50-60 [7]. GPA is a rare rheumatologic disorder in Iranian children with prevalence less than 1 per million children [8].

Etiology still remains unknown, but it is usually associated with antineutrophil cytoplasmic antibody (ANCA) that was detected in predominantly cytoplasmic form (cANCA). Granulomatous lesions of the upper and lower airways and the kidneys are predominant features of the disease [9].

GPA is a very rare condition in pediatric population, despite the fact that it appears to be the same disease observed in adults, clinical presentations vary in both population. In children, high frequency of constitutional symptoms, upper and lower respiratory tract, mucosa, and skin and musculoskeletal involvement could be considered as characteristics of the disease based on an analysis of 56 affected children [5].

It has long been known that kidney and lung involvement are major predictors of mortality, and cardiac involvement is relatively infrequent in GPA [10, 11]. In one study of all patients with GPA diagnosis who were referred to all Iranian Pediatric Rheumatology Division from 2002 to 2011, in total of 11 patients, the most common organ system involvement was upper respiratory tract (81.8%), and lower respiratory tract involvement was seen in 63.9%. Six children (54.4%) had abnormal imaging in chest X-ray or chest CT-scan. Renal involvement in 4 children (36.3%) and venous thrombosis in 1 patient (9%) were observed [8].

In one cohort of 158 patients with GPA, cardiac manifestations (predominantly pericarditis) were present in 10 patients (6%), and, of these, 3 patients had coronary artery involvement [12].

Although the leading cause of coronary aneurysms is atherosclerosis, it also could be seen as an uncommon feature among some connective tissue disorders. Kawasaki disease (KD) and polyarthritis nodosa (PAN) constitute most cases of coronary aneurysms in the presence of connective tissue diseases; however, other small vessel vasculitis can also develop relatively large aneurysms [13]. Hence, GPA, classified as small to medium vessel vasculitis which is associated with positive ANCA titer, is capable of evolving aneurysms in branches of renal, hepatic, gastric, splanchnic, subclavian, and cerebral most commonly with some rare incidence of affecting walls of coronary arteries [14, 15]. Moreover, thrombosis is not frequently associated with GPA yet was reported to occur commonly at large and small intracranial vessels, deep veins of extremities, sinuses, lungs, and kidneys. The presence of any systemic predisposition for hypercoagulability state is still a debating subject [16]. In the most cases of GPA, atherosclerotic vascular disease, particularly that seen in systemic lupus erythematous (SLE) and rheumatoid arthritis (RA), has not been documented, reflecting the fact that vascular inflammation and thrombosis, including coronaritis and coronary thromboembolism, as more characteristics for GPA [17–19].

In our case, the onset of the disease with recurrent upper and lower respiratory infections was frequent and had deep vein thrombosis (DVT) in the course of the disease.

The association between respiratory infection and risk of heart attacks and strokes is well established. However, less evidence exists for an association between respiratory infection and venous thromboembolism (VTE) and probably is an increased risk following infection for pericardial effusion (PE) as well as for DVT and that this may be related to the severity of the infection [20].

Another diagnosis initially given to the patient due to fever and evidence of coronary involvement (diffuse ectasia and dilation with huge aneurysm in the left anterior descending artery with the size of 14 mm) was KD.

Kawasaki disease (KD) is a vasculitis of medium-sized vessels the majority of cases occurs in children under 5 years of age. The dreaded cardiac complication is coronary artery involvement, present in 20% of cases, resulting in myocardial infarction and aneurysm formation. Hence, KD should be considered as the possible cause of acute coronary syndromes in young adults, particularly when aneurysms are found. In the absence of generalized atherosclerotic disease, a history of KD-like illness in childhood should always be sought in these cases [6].

KD and PAN are the systemic vasculitis that more often present coronary artery aneurysms [6]. Mavrogeni et al. using magnetic resonance angiography showed discrete fusiform coronary aneurysms in patients with PAN and microscopic polyangiitis, but they did not find them in patients with GPA or eosinophilic GPA (Churg-Strauss syndrome) [21].

Relapsing polychondritis (RP) is another cause of coronary artery aneurysm that frequently has saddle-nose deformity as an otolaryngological manifestation [22]; 24% of these patients could present low titles of ANCA, especially P-ANCA, that showed myeloperoxidase antigen specificity [23].

In our case, patient's age, the absence of aneurysms in the kidneys and abdomen, and positive ANCA C with high levels of proteinase-3 suggested that the coronary artery aneurysms observed were caused by diseases other than RP, KD, or PAN, such as ANCA-associated vasculitis, in particular, GPA.

Finally, GPA was diagnosed for him based on history of severe upper airway infection, saddle nose, ANCA and P-ANCA positivity, and cavitary pulmonary lesion. Thrombosis occurred within the enlarged space, 8 months after salicylate administration which required high-dose methylprednisolone application for three subsequent days, 10 mg/kg of heparin, and warfarin maintenance therapy to reach INR level of 3. The mass resembling thrombosis at the echocardiographic study was reduced by 10 days following mixed anticoagulation therapy and resolved 3 months later.

The risk of thromboembolic events increases in patients with WD [24], but pulmonary hypertension is exceptionally described up till now [25]. Atherosclerosis is the main cause of coronary artery aneurysm; however, they can be observed in connective tissue diseases such as systemic lupus erythematosus and vasculitis. KD and polyarteritis nodosa (PAN) are the systemic vasculitis that more often present coronary artery aneurysms.

There are descriptions in the literature that small vessel vasculitis such as microscopic polyangiitis and PAN could develop coronary artery aneurysm, which are infrequent in other ANCA-associated vasculitis. Musuruana et al. report a 25-year-old with WG and extensive anterior myocardial infarction. Coronary artery aneurysms were shown in cardiac angiography and were resolved after treatment with high doses of corticosteroids and cyclophosphamide [13].

In conclusion, coronary aneurysms and vascular thrombosis are rare presentations of GPA. So, GPA should be considered as differential diagnosis in patients with prolong fever and coronary aneurysms as atypical Kawasaki disease in children and adolescents. We suggest that the desirable therapeutic management of aneurysm of GPA lies in administering the combination of ASA and warfarin, as initial treatment even though no giant coronary aneurysms are detectable with the presence of merely small-sized aneurysms so to prevent consecutive thrombotic complication.
